# Supplementary material for: Intrinsic electrical activity drives small-cell lung cancer progression
Source: Nature. 2025 Feb 12;639(8055):765–75. doi: 10.1038/s41586-024-08575-7 (PMC11922742; doi:10.1038/s41586-024-08575-7)

nature portfolio

https://doi.org/10.1038/s41586-024-08575-7

Supplementary information

Intrinsic electrical activity drives small-cell lung cancer progression

In the format provided by the
authors and unedited

Fig. 3k)

Fig. 4i)

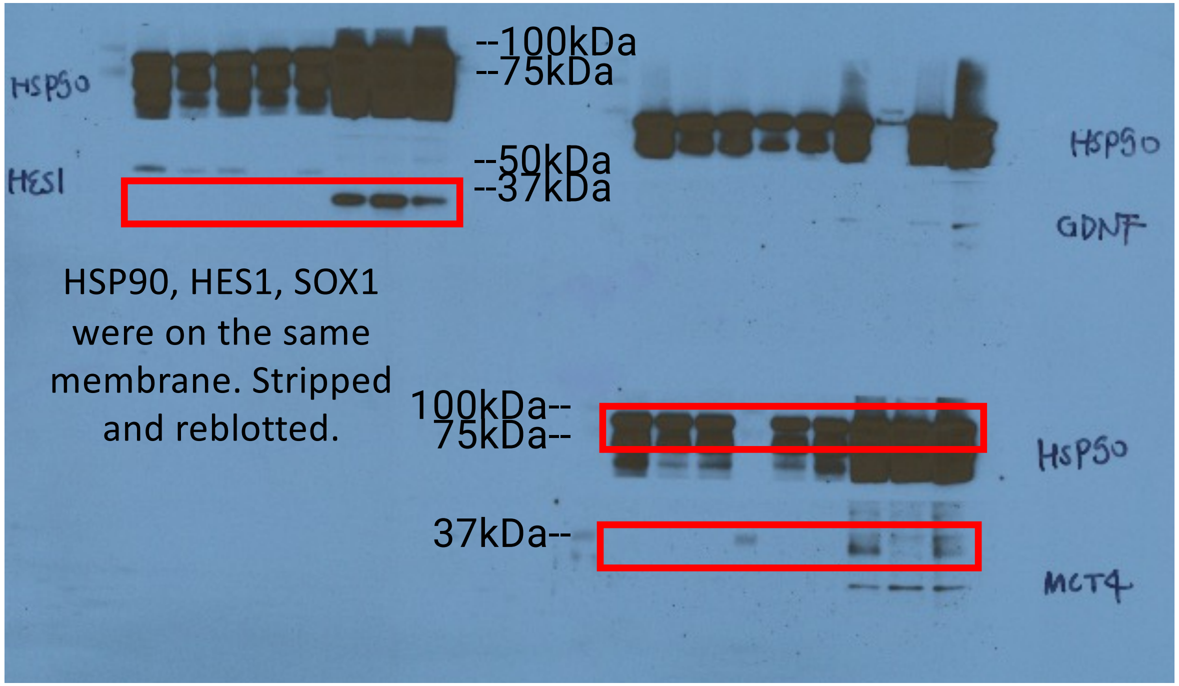

HSP90 and MCT4 were on the same membrane. Each at different exposure.

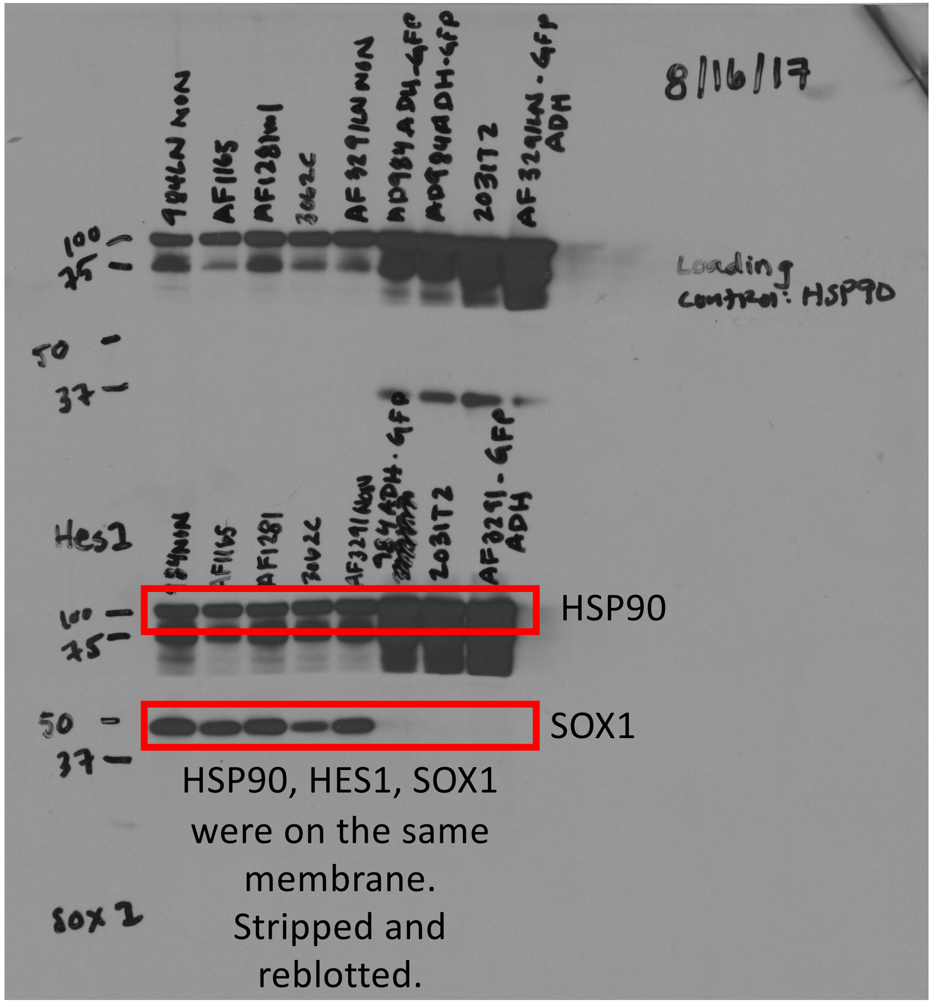

Extended Data Fig.7f) **FLAG blot**

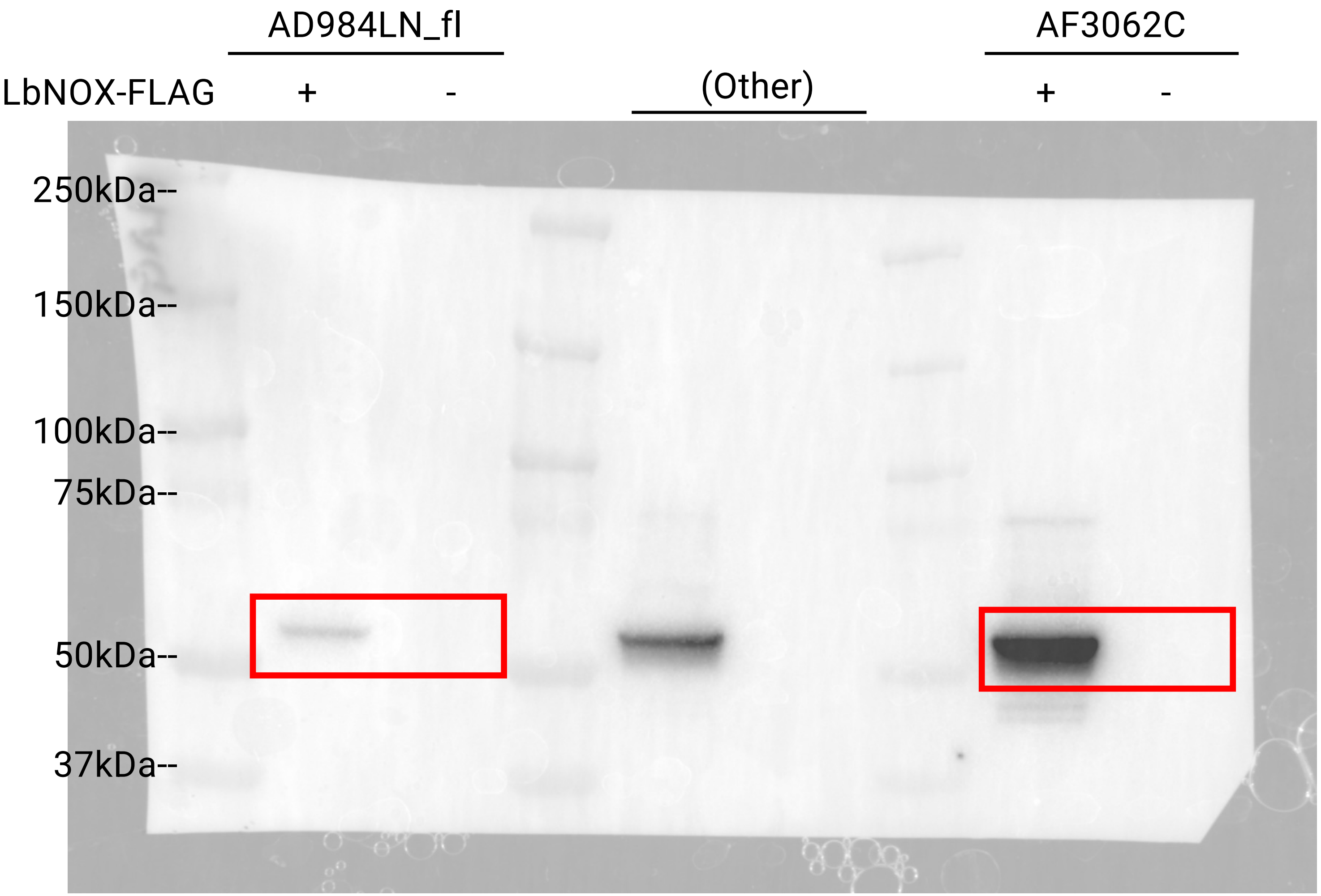

Extended Data Fig.7f) **αTubulin reblot after HRP-quenching with H_2O_2 (FLAG membrane)**

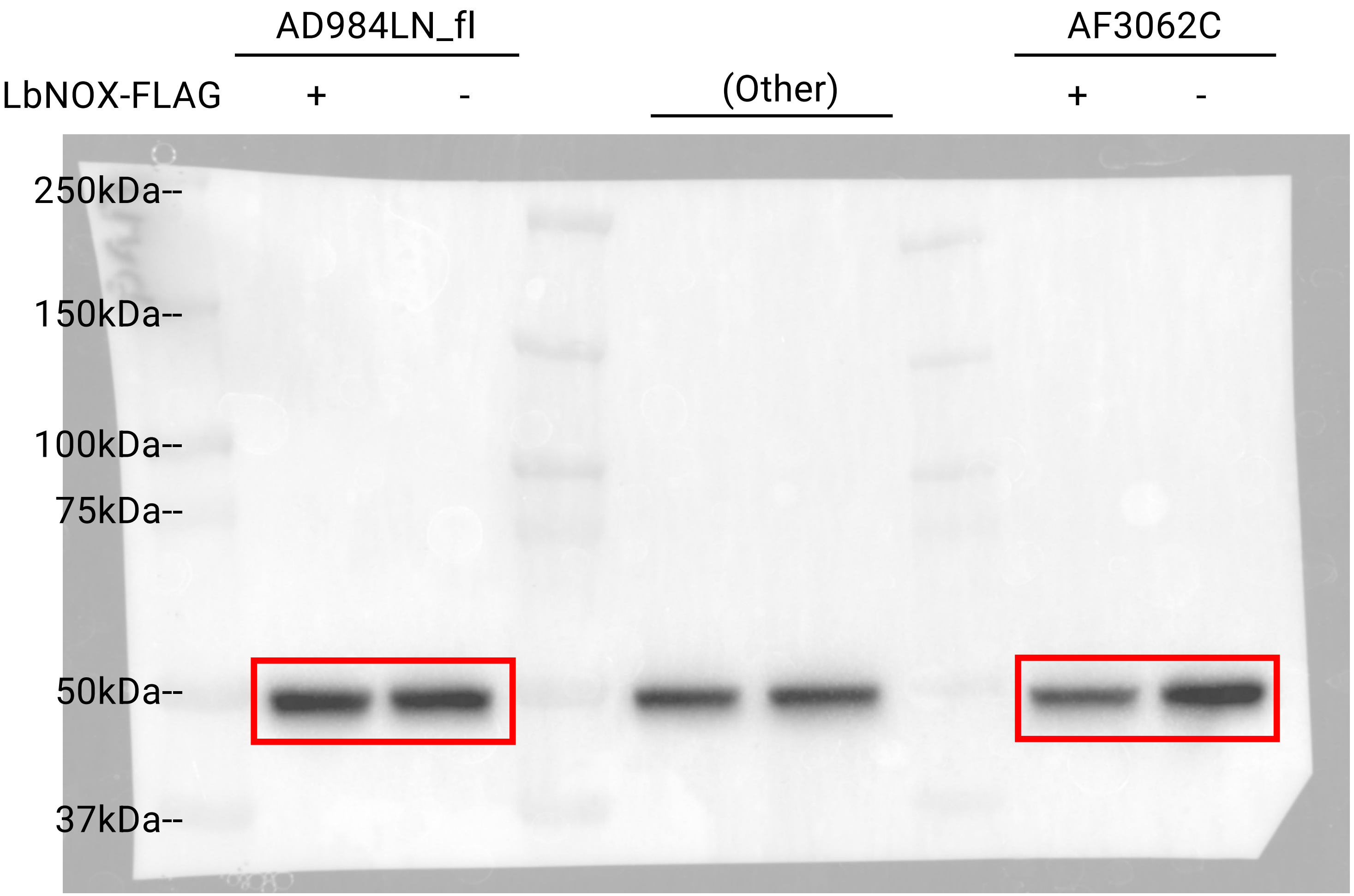

Extended Data Fig.7p) **MCT1 and αTubulin (reblot)**

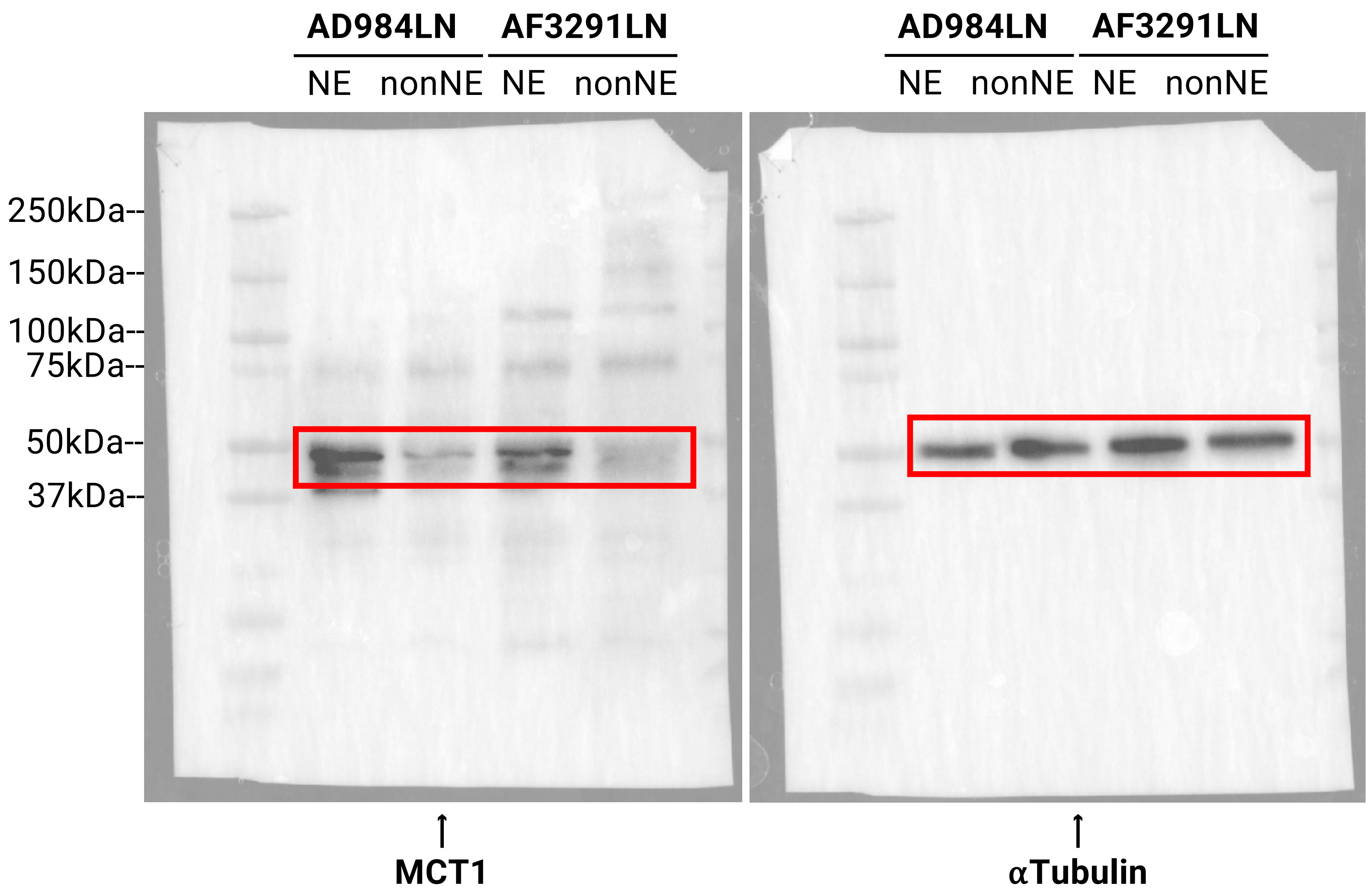

Extended Data Fig.9g) **C-FOS blot**

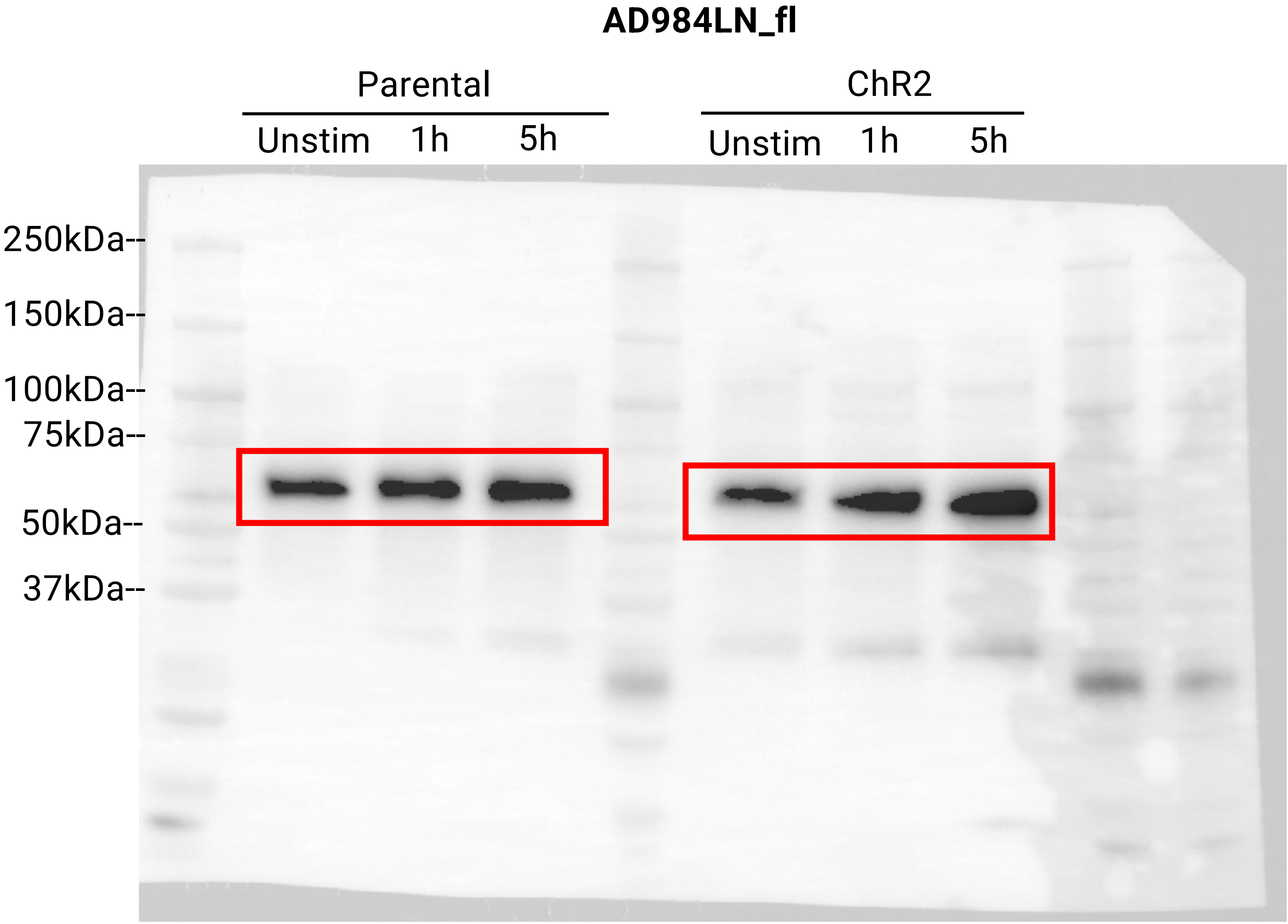

Extended Data Fig.9g) **αTubulin reblot (c-FOS membrane)**

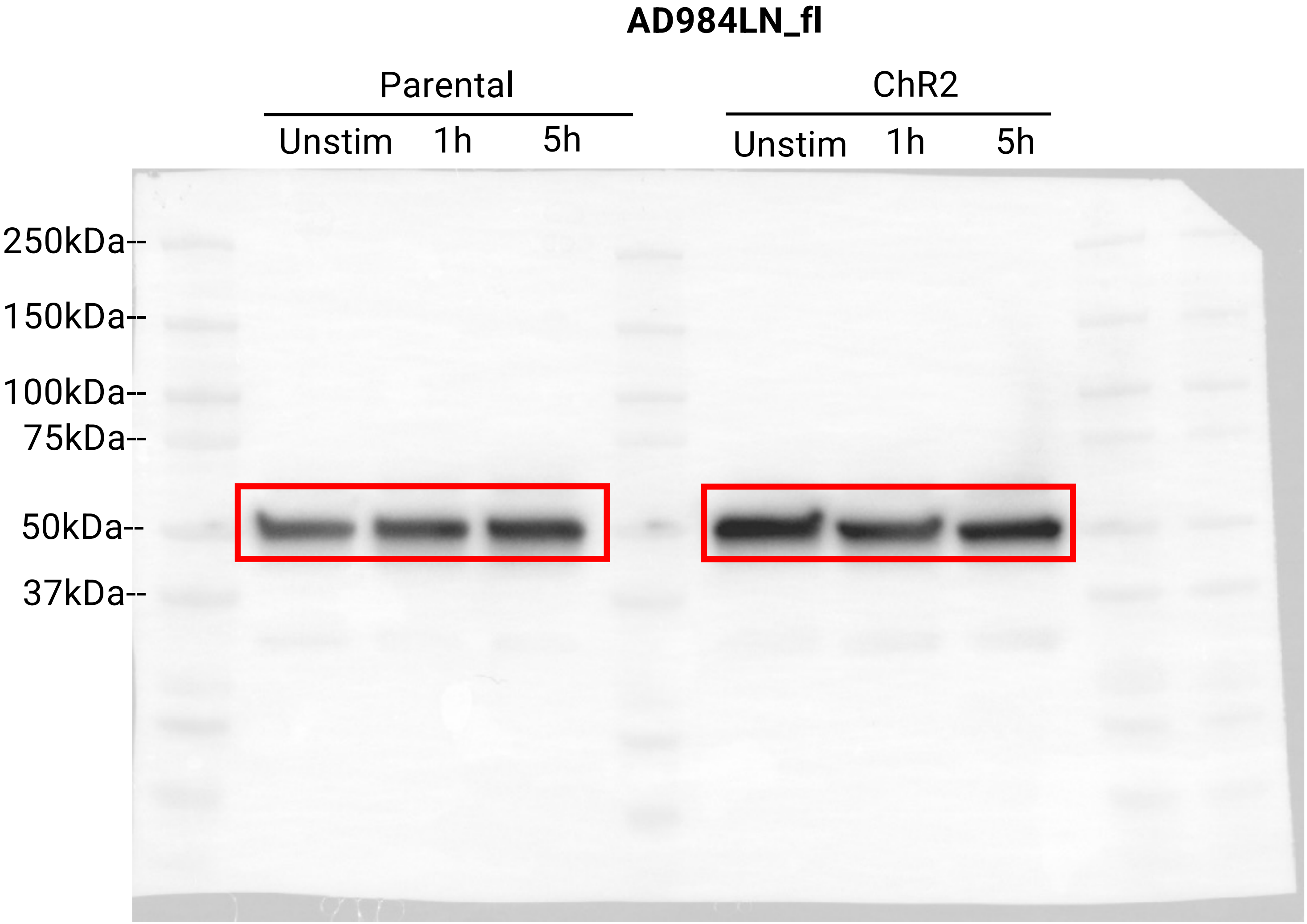

Extended Data Fig.9g) **p-CREB blot**

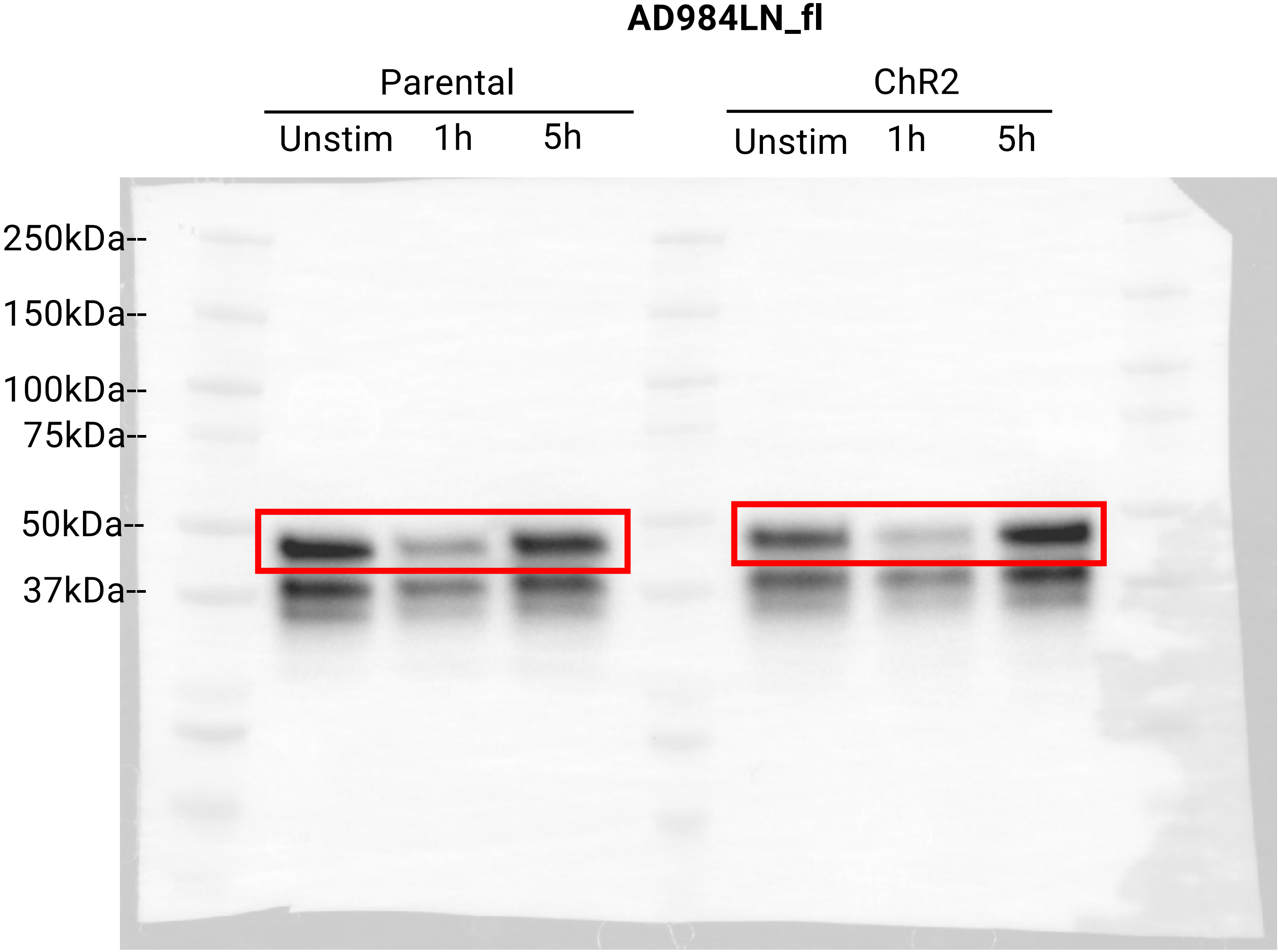

Extended Data Fig.9g) **αTubulin reblot (p-CREB membrane)**

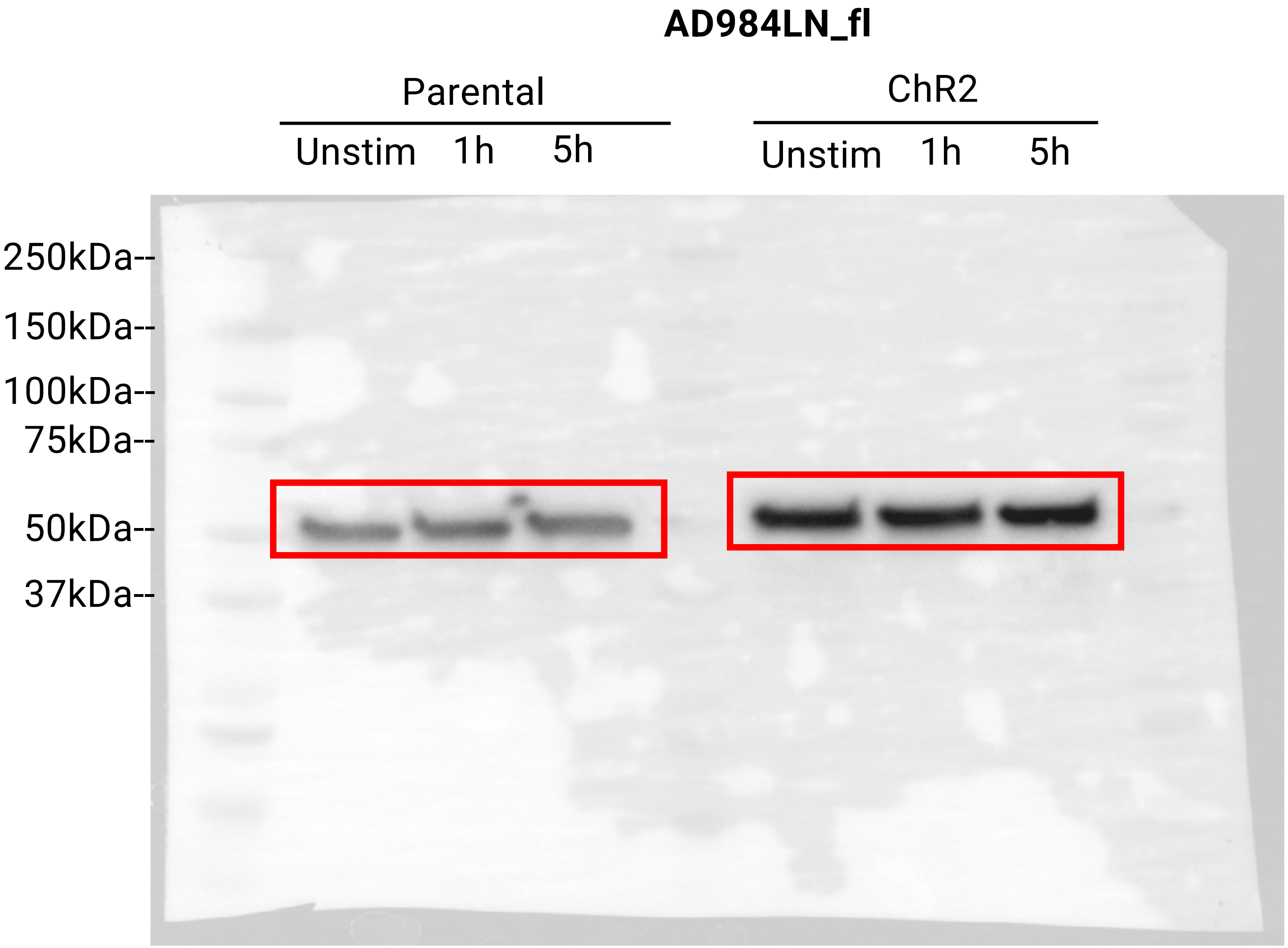

Extended Data Fig.9g) **C-FOS blot**

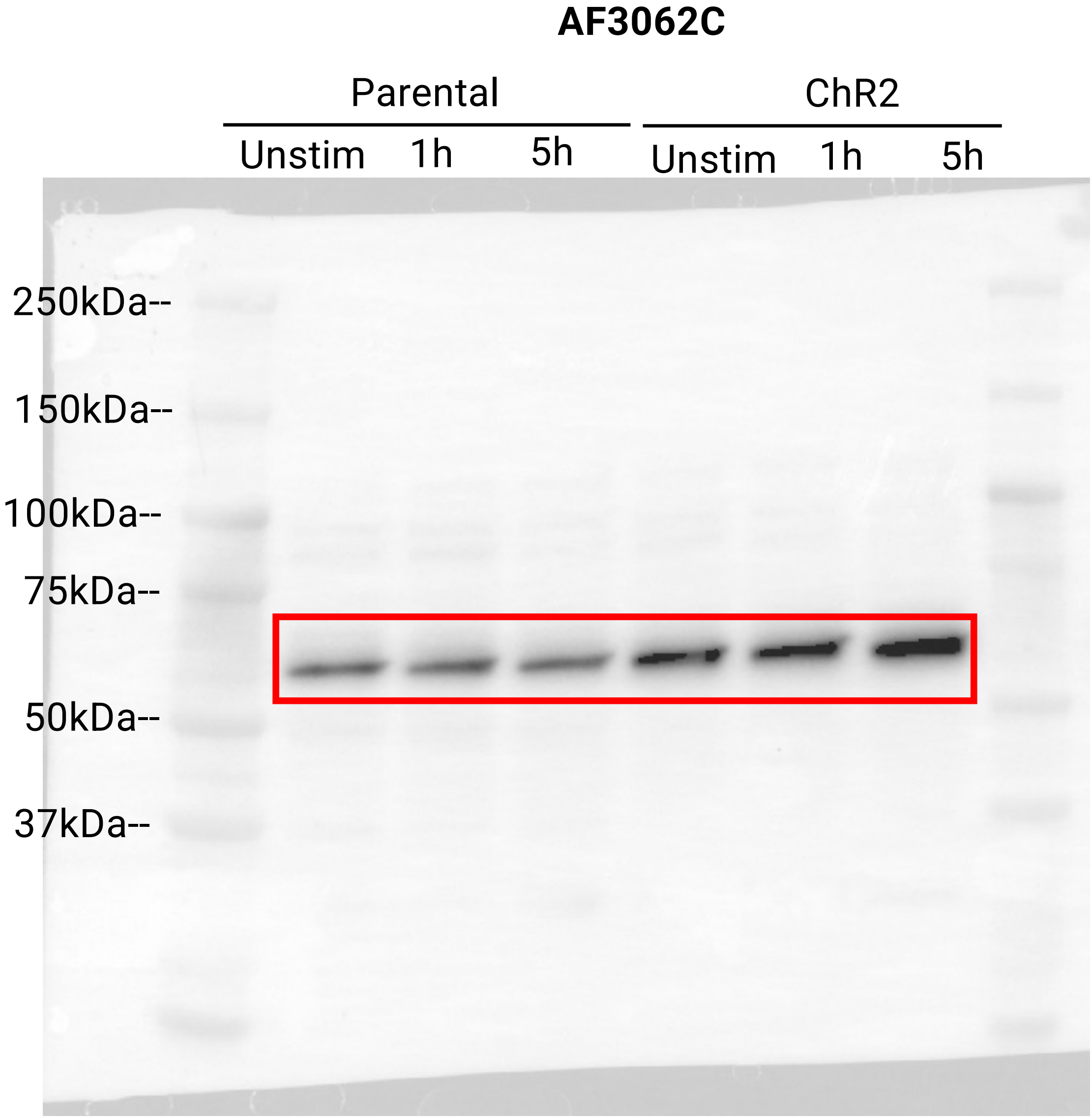

Extended Data Fig.9g) **αTubulin reblot (c-FOS membrane)**

AF3062C

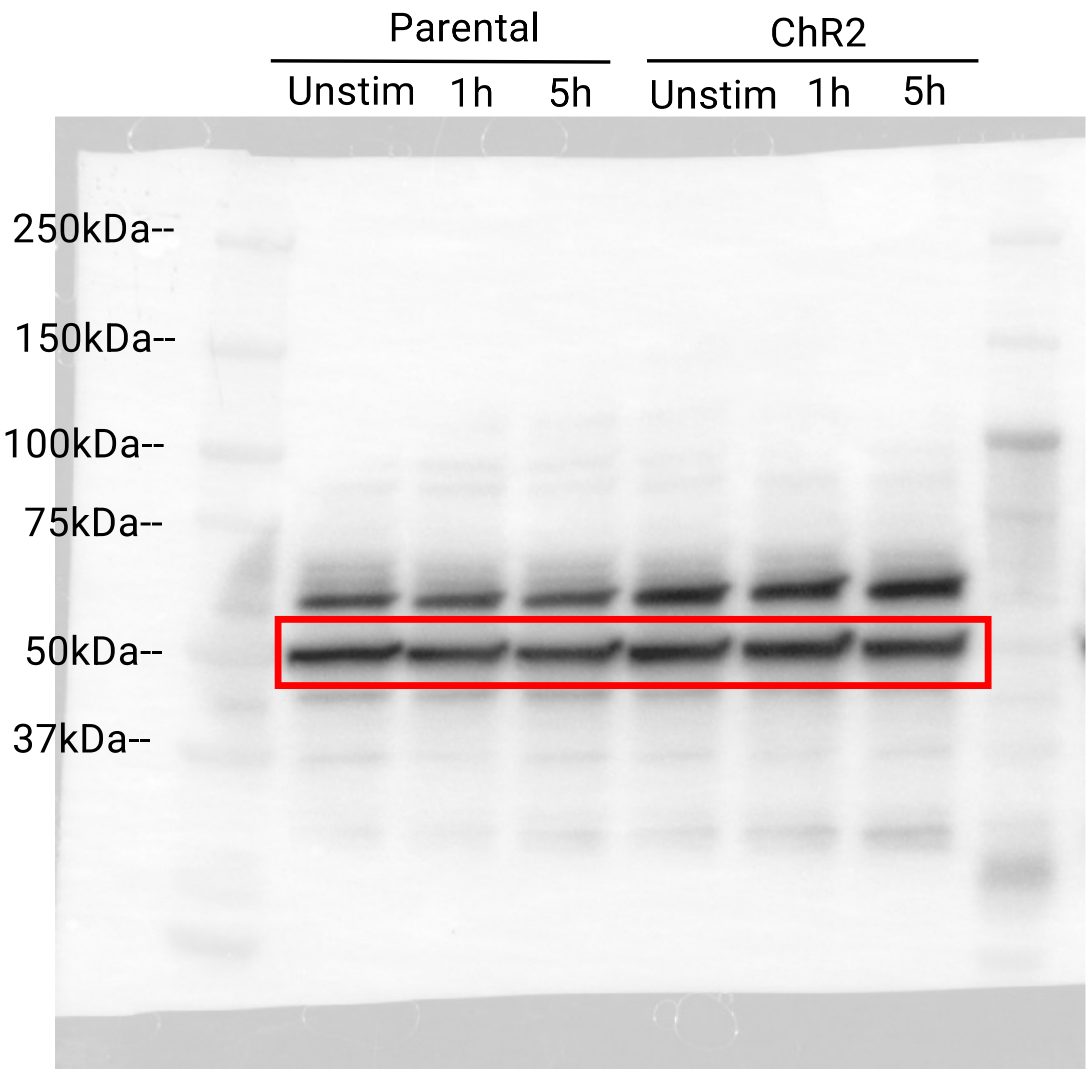

Extended Data Fig.9g) **p-CREB blot**

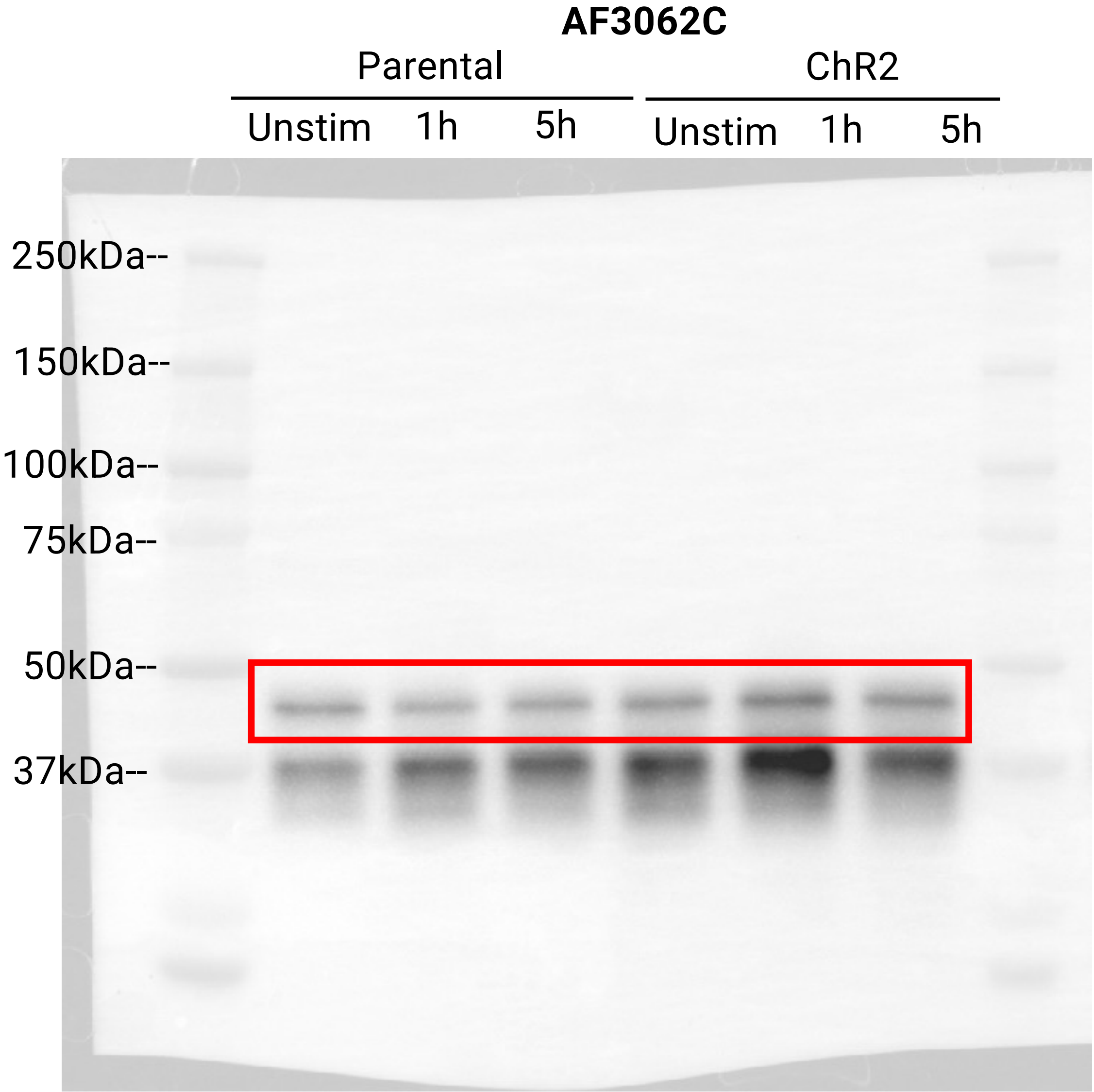

Extended Data Fig.9g) **αTubulin reblot (p-CREB membrane)**

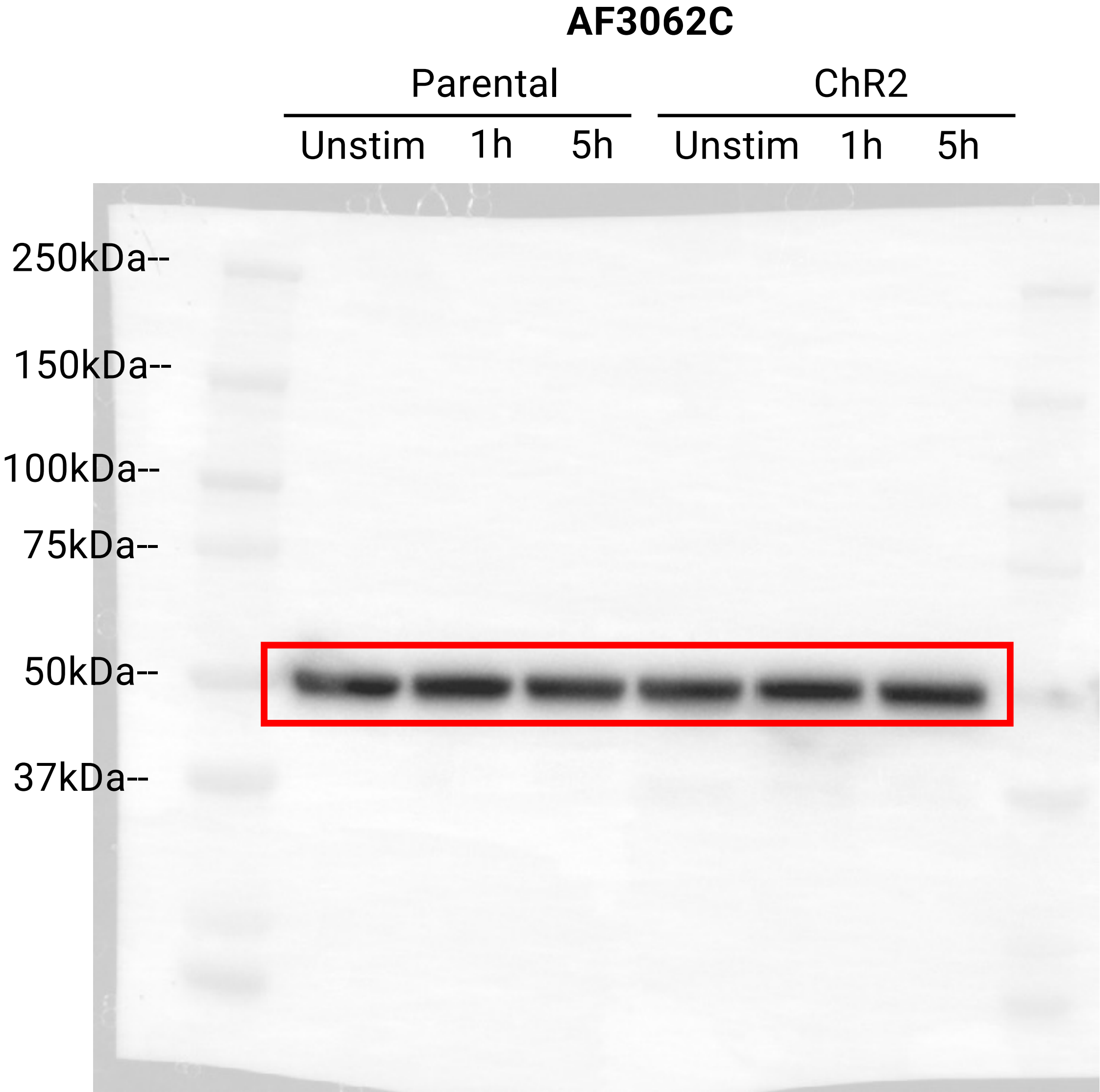

Extended Data Fig.9k) **4-HNE and αTubulin (reblot)**

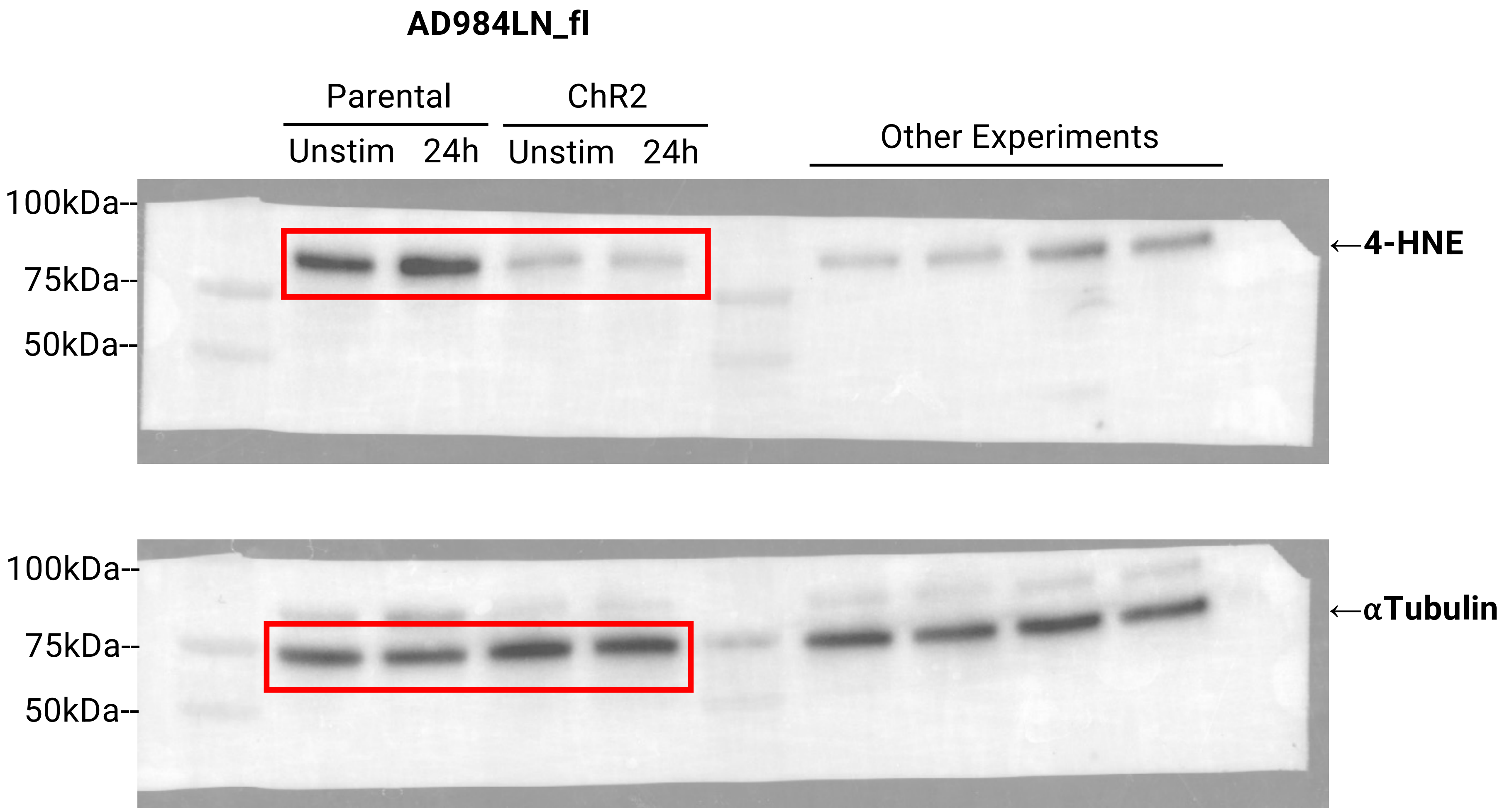

Extended Data Fig.9k) **LCB3 and αTubulin (reblot)**

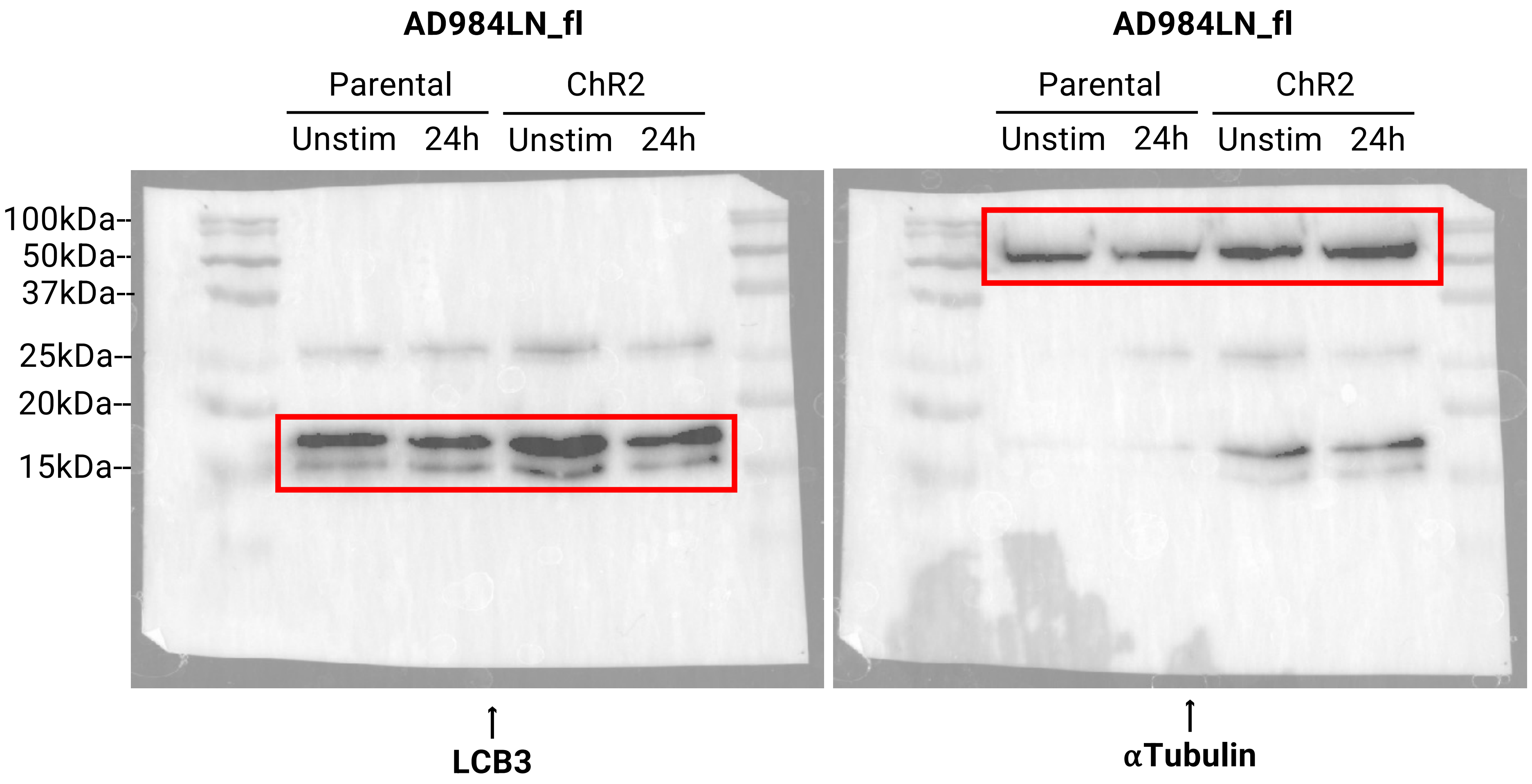

Extended Data Fig.9l) **GPX4 and αTubulin**

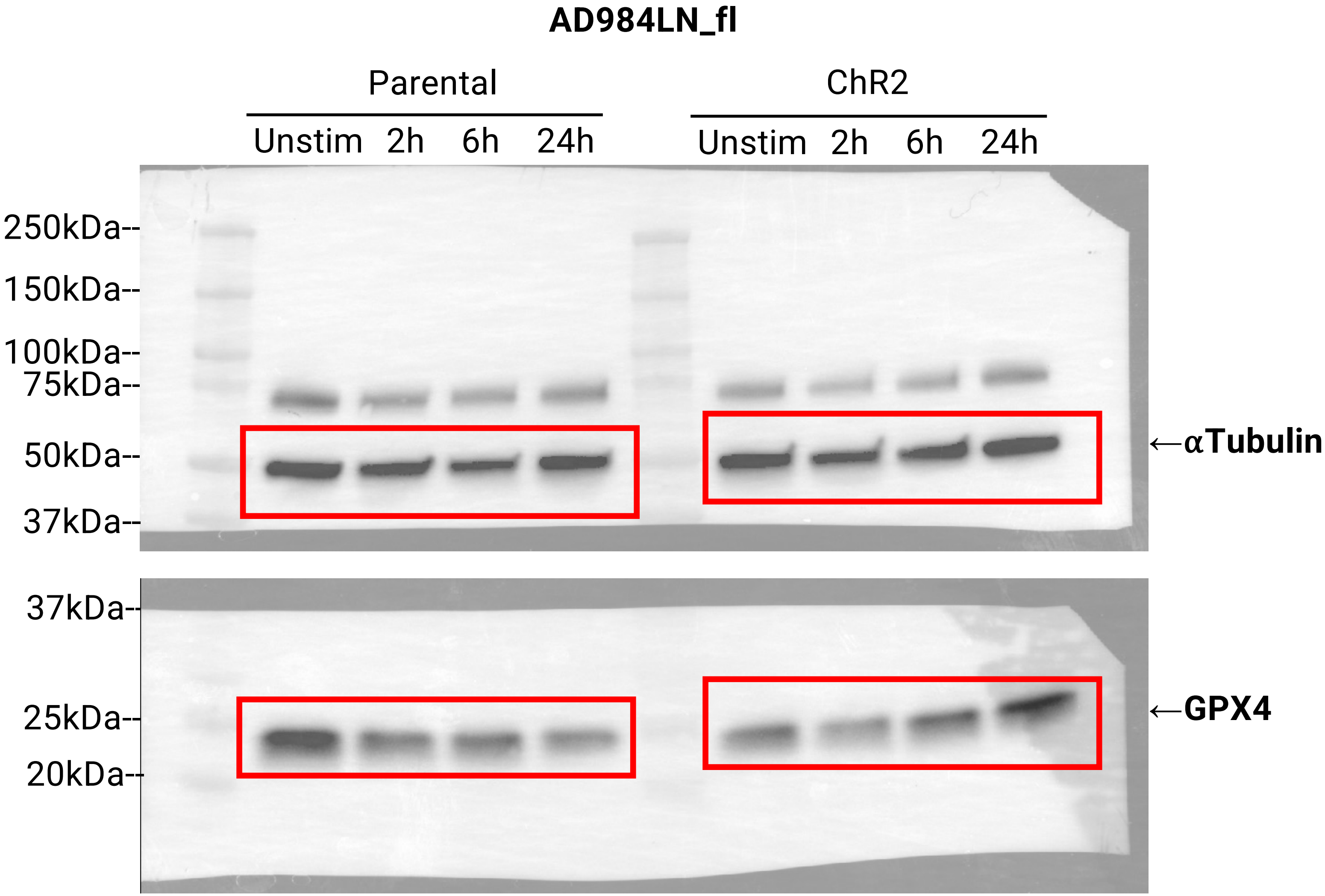

Extended Data Fig.10d) **P-CREB blot**

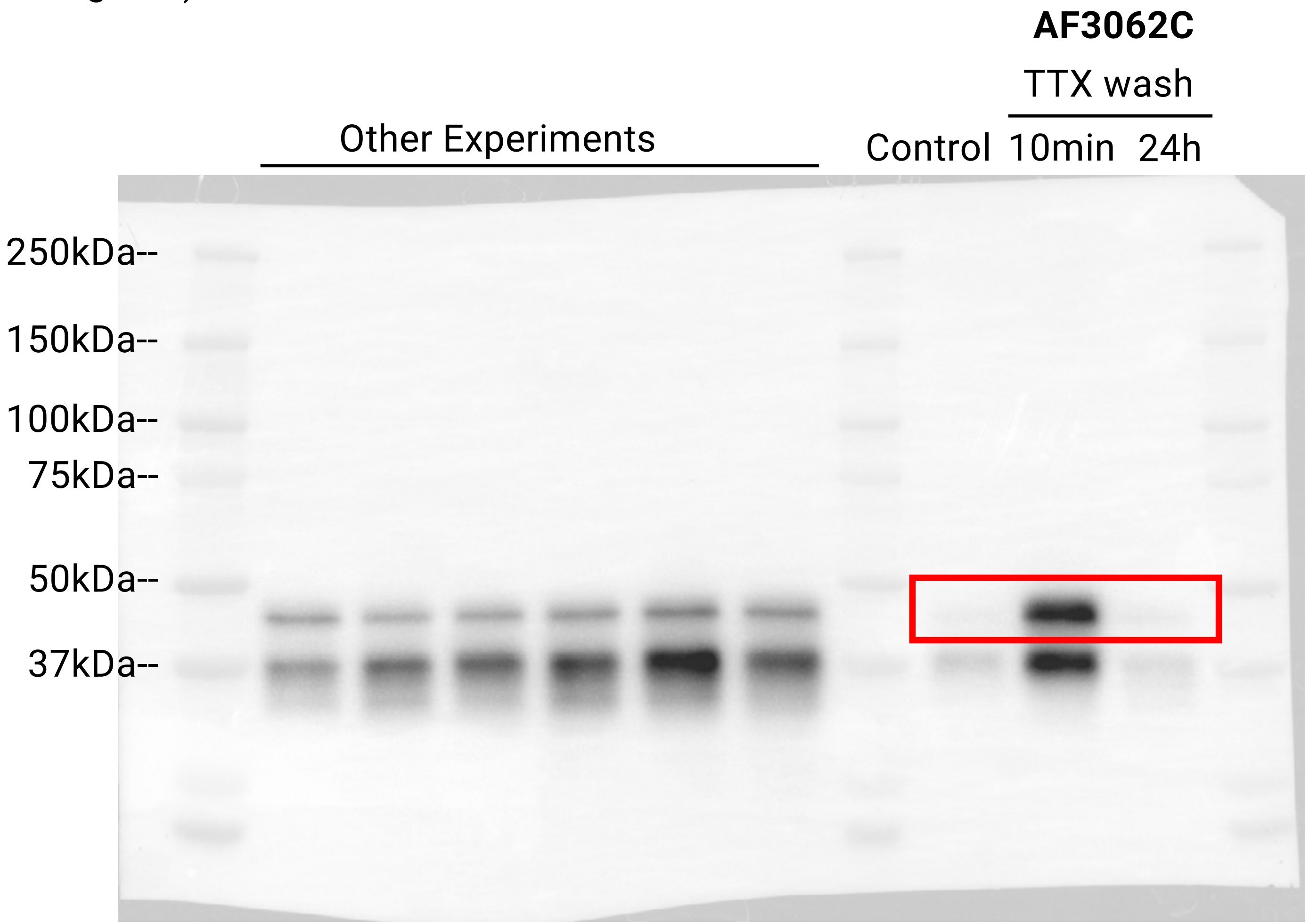

Extended Data Fig.10d) **αTubulin reblot (p-CREB membrane)**

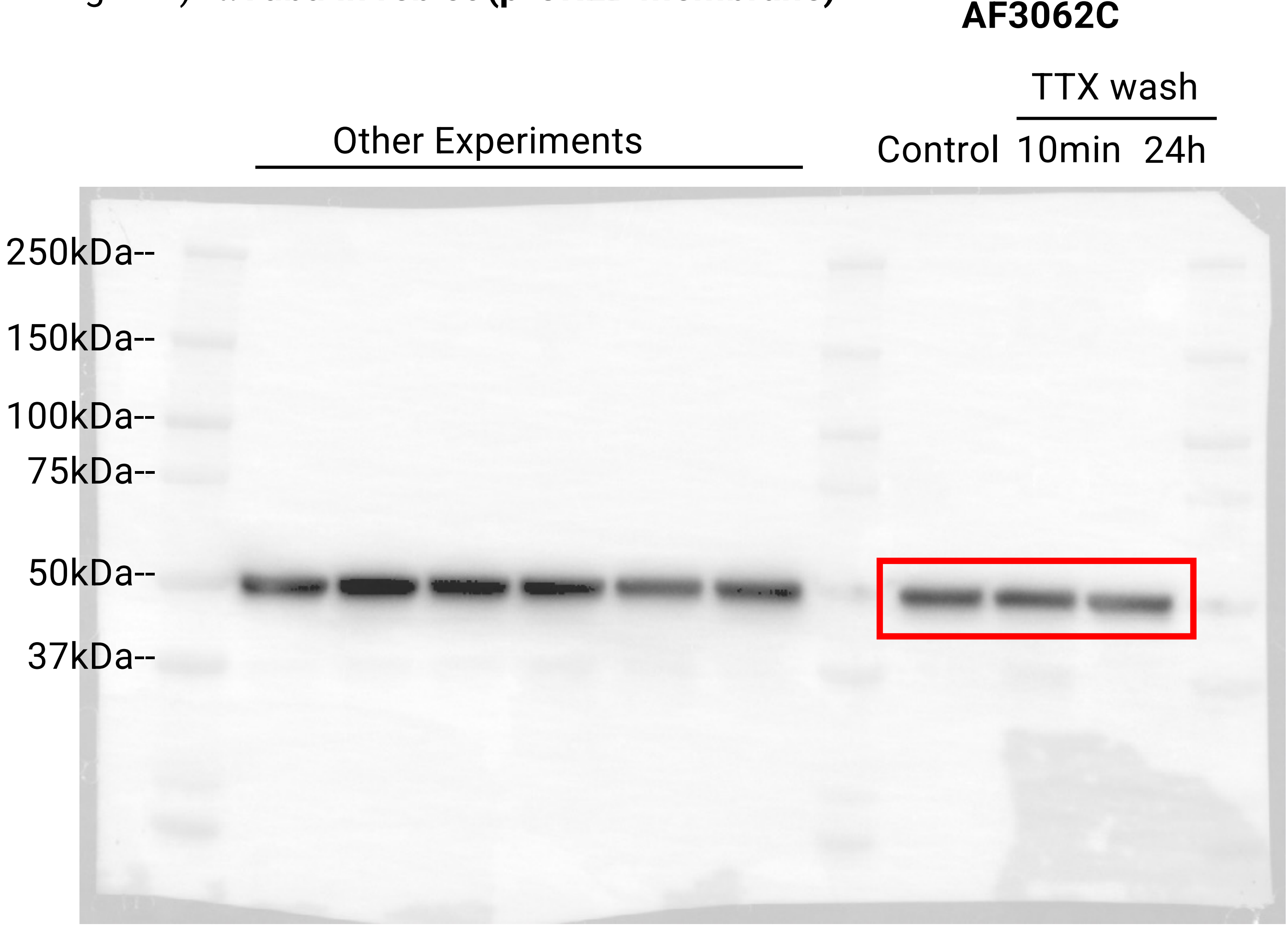

Supplement: Supplementary file 1 — Uncropped and unprocessed western blot images. [file 41586_2024_8575_MOESM1_ESM.pdf]
