# Supplementary material for: Intrinsic electrical activity drives small-cell lung cancer progression
Source: Nature. 2025 Feb 12;639(8055):765–75. doi: 10.1038/s41586-024-08575-7 (PMC11922742; doi:10.1038/s41586-024-08575-7)
Supplement: Supplementary file 2 — Reporting Summary [file 41586_2024_8575_MOESM2_ESM.pdf]

nature portfolio

2023-10-17704D
Corresponding author(s): Leanne Li
Last updated by author(s): Nov 11, 2024

Reporting Summary

Nature Portfolio wishes to improve the reproducibility of the work that we publish. This form provides structure for consistency and transparency in reporting. For further information on Nature Portfolio policies, see our Editorial Policies and the Editorial Policy Checklist.

Statistics

For all statistical analyses, confirm that the following items are present in the figure legend, table legend, main text, or Methods section.

n/a	Confirmed	
☐	☒	The exact sample size (*n*) for each experimental group/condition, given as a discrete number and unit of measurement
☐	☒	A statement on whether measurements were taken from distinct samples or whether the same sample was measured repeatedly
☐	☒	The statistical test(s) used AND whether they are one- or two-sided<br>*Only common tests should be described solely by name; describe more complex techniques in the Methods section.*
☒	☐	A description of all covariates tested
☐	☒	A description of any assumptions or corrections, such as tests of normality and adjustment for multiple comparisons
☐	☒	A full description of the statistical parameters including central tendency (e.g. means) or other basic estimates (e.g. regression coefficient) AND variation (e.g. standard deviation) or associated estimates of uncertainty (e.g. confidence intervals)
☐	☒	For null hypothesis testing, the test statistic (e.g. F, t, r) with confidence intervals, effect sizes, degrees of freedom and P value noted<br>*Give P values as exact values whenever suitable.*
☒	☐	For Bayesian analysis, information on the choice of priors and Markov chain Monte Carlo settings
☒	☐	For hierarchical and complex designs, identification of the appropriate level for tests and full reporting of outcomes
☐	☒	Estimates of effect sizes (e.g. Cohen's *d*, Pearson's *r*), indicating how they were calculated

Our web collection on statistics for biologists contains articles on many of the points above.

Software and code

Policy information about availability of computer code

Data collection	MicroManager open-source software (version 2.0.0) was used for acquisition of calcium imaging data.<br>For the RNA-seq dataset, Illumina HiSeq 2000 50-nt single-ended reads were mapped to the UCSC mm9 mouse genome build (http://genome.ucsc.edu/) using rsem v1.2.12 and bowtie v1.0.1 with default options.<br>For in vivo bioluminescent imaging, Living Image software 4.8 has been used.
Data analysis	-Biophysical modelling of ATP consumption: A custom script in Matlab R2022b Update! (9.13.0.2080170) was used following the model described in the M&M section and can be accessed at https://doi.org/10.6084/m9.figshare.27630099<br>-Calcium Imaging Analysis: ImageJ (version 1.54f) with TrackMate plugin and Cellpose detector was used for image segmentation, tracking of cells and outputing fluorescence intensity measurements for segmented cells at each time point analyzed.<br>A custom script in Matlab R2022b Update! (9.13.0.2080170) was used for finding calcium peaks and outputing peak frequency and peak prominence. The Matlab code for calcium imaging analyses can be accessed at https://doi.org/10.6084/m9.figshare.27630099<br>-RNA-seq analysis: Raw estimated expression counts were upper-quartile normalized to a count of 1000. Given the complexity of the dataset in terms of a mixture of different biological backgrounds, a high-resolution signature discovery approach was employed to characterize global gene expression profiles. Independent Component Analysis (ICA), an unsupervised blind source separation technique, was used on this discrete count-based expression dataset to elucidate statistically independent and biologically relevant signatures as detailed in the M&M section. All RNA-seq analyses were conducted in the R Statistical Programming language (v.3.6.0). Gene set enrichment analysis (GSEA) was carried out using the pre-ranked mode with default settings. Heatmaps were generated using the Heatplus package in R (v.2.26.0).<br>-Graph Pad Prism has been employed for statistical analysis (v. 10.3.0).<br>-In vivo bioluminescent imaging: Living Image software 4.8 has been used.<br>-Immunofluorescence analysis: ImageJ (version 1.54f) with neuronj plugin has been used for cumulative axon length quantification; JaCoP plugin has been employed to calculate the Pearson's correlation coefficient. Qupath 0.5.0 has been used for TMA and IHC quantification.

Imaris has been used for 3D rendering and visualization (v. 10.1.1).
-Oxygen consumption rates and downstream analyses were performed using Seahorse Analytics (Version 1.0.0-699).

For manuscripts utilizing custom algorithms or software that are central to the research but not yet described in published literature, software must be made available to editors and reviewers. We strongly encourage code deposition in a community repository (e.g. GitHub). See the Nature Portfolio guidelines for submitting code & software for further information.

Data

Policy information about availability of data

All manuscripts must include a data availability statement. This statement should provide the following information, where applicable:

- Accession codes, unique identifiers, or web links for publicly available datasets
- A description of any restrictions on data availability
- For clinical datasets or third party data, please ensure that the statement adheres to our policy

Raw and processed data of SCLC and PDAC cell lines from the RNA-seq experiments have been deposited in the NCBI Gene Expression Omnibus database (accession number: GSE270281). RNA-seq data of LUAD cell lines have been deposited under the accession code GSE14594584. Expression data from human SCLC cell lines was accessed from the Cancer Dependency Portal (DepMap) (www.depmap.org), specifically from the DepMap Public 23Q2 and the proteomic dataset. The values from this the DepMap Public 23Q2 dataset are inferred from RNA-seq data using the RSEM tool and are reported after log2 transformation, using a pseudo-count of 1; log2 (TPM+ 1). Clinical and expression data from a cohort of 112 SCLC patients were obtained from Liu et al, Cell 187, 184-203 e128 (2024). Expression data and NE Scores from patient-derived xenografts (PDX) models were provided by Dr. Benjamin J. Drapkin.

Research involving human participants, their data, or biological material

Policy information about studies with human participants or human data. See also policy information about sex, gender (identity/presentation), and sexual orientation and race, ethnicity and racism.

Reporting on sex and gender	NA
Reporting on race, ethnicity, or other socially relevant groupings	NA
Population characteristics	NA
Recruitment	NA
Ethics oversight	NA

Note that full information on the approval of the study protocol must also be provided in the manuscript.

Field-specific reporting

Please select the one below that is the best fit for your research. If you are not sure, read the appropriate sections before making your selection.

☒ Life sciences ☐ Behavioural & social sciences ☐ Ecological, evolutionary & environmental sciences

For a reference copy of the document with all sections, see nature.com/documents/nr-reporting-summary-flat.pdf

Life sciences study design

All studies must disclose on these points even when the disclosure is negative.

Sample size	No statistical methods were used to calculate sample size. Sample sizes were chosen based on preliminary experiments aiming to capture the biological effects in line with similar research in the field.
Data exclusions	No data have been excluded from the analysis.
Replication	The number of replicates for each experiment has been reported in the corresponding figure legend and methods. RNA-seq experiments were performed in 5 or 3 different cell lines for the mSCLC-NE or non-NE group, respectively.
Randomization	For cell culture works, samples were randomly assigned to the experimental groups. For in vivo work, age-, litter-, and sex-matched mice were randomly assigned to experimental groups. For imaging analyses, fields of view were randomly selected and when required, allocated to the different experimental groups based on the size of the tumor or the lack thereof. For electrophysiology experiments, cells were randomly selected for subsequent analyses. For human Tissue Microarray (TMA), the whole biopsy sections were analyzed and classified according to their histological stage as provided by the supplier.
Blinding	We ensured blinded conduct during data acquisition and the subsequent analysis. When the experimental setup allows it, such as in the intrasplenic injection of cancer cells with different treatments, the experiment was conducted by a researcher who was blind to the experimental groups.

Reporting for specific materials, systems and methods

We require information from authors about some types of materials, experimental systems and methods used in many studies. Here, indicate whether each material, system or method listed is relevant to your study. If you are not sure if a list item applies to your research, read the appropriate section before selecting a response.

Materials & experimental systems

n/a	Involved in the study
☐	☒ Antibodies
☐	☒ Eukaryotic cell lines
☒	☐ Palaeontology and archaeology
☐	☒ Animals and other organisms
☒	☐ Clinical data
☒	☐ Dual use research of concern
☒	☐ Plants

Methods

n/a	Involved in the study
☒	☐ ChIP-seq
☒	☐ Flow cytometry
☒	☐ MRI-based neuroimaging

Antibodies

Antibodies used

Primary Antibodies (dilutions reported in Methods):
MCT4 (Santa Cruz, sc-376140)
SOX1 (Bio-Techne Ltd, AF3369)
VAChT (Synaptic System, 139105)
TdTomato (Sicgen, AB8181-200)
β3 tubulin (Abcam, ab52623)
Phospho-Ser133-CREB (Ser133) (Cell Signaling, 9198)
c-FOS (Abcam, ab190289)
alpha-Tubulin-HRP (Abcam, ab40742)
HES1 (Cell Signaling, 11988S)
MCT1 (Sigma, ab1286-I)
SOX1 (Cell Signaling, 4194)
GPX4 (Abcam, ab125066)
4-hne (Abcam, ab46545)
LC3B (Cell Signaling, 43566)
hsp90 (BD, 610418)
FLAG (Cell Signaling, 2368)
Ki67 (Abcam, ab15580)
Cleaved Caspase 3 (Cell Signaling, 9579)

Secondary antibodies (dilutions reported in Methods):
Goat anti-rabbit HRP-conjugated (Abcam, ab205718)
Goat anti-rmouse HRP-conjugated invitrogen g-21040
Goat anti-chicken HRP-conjugated invitrogen a16054
Donkey anti-mouse Alexa Fluor (AF) 488 (Invitrogen, A21202)
Donkey anti-rabbit AF 488 (Invitrogen, A-21206)
Donkey anti-goat AF 568 (Invitrogen, A-11057)
Donkey anti-goat AF 647 (Invitrogen, A32849)
Goat anti-guinea pig AF 647 (Invitrogen, A21450)

Validation

Primary antibodies:
-MCT4 (Santa Cruz, sc-376140) previously validated in GEMM models in the following manuscript (Qian Y et al. (2023). MCT4-dependent lactate secretion suppresses antitumor immunity in LKB1-deficient lung adenocarcinoma. Cancer Cell 41(7):1363-1380.e7. doi: 10.1016/j.ccell.2023.05.015)
-SOX1 (Bio-Techne Ltd, AF3369) previously validated in mouse cortical stem cells as stated by the manufacturers on their website (https://www.bio-techne.com/p/antibodies/human-mouse-rat-sox1-antibody_af3369)
-VAChT (Synaptic System, 139105) previously validated in mouse spinal cord section as stated by the manufacturers on their website (https://sysy.com/product/139105)
-TdTomato (Sicgen, AB8181-200) previously validated in 293HEK cells transfected with cDNA as stated by the manufacturers on their website (https://www.origene.com/catalog/antibodies/tag-antibodies/ab8181-200/tdtomato-goat-polyclonal-antibody)
-β3 tubulin (Abcam, ab52623) previously validated in human cerebellum tissue section as stated by the manufacturers on their website (https://www.abcam.com/products/primary-antibodies/beta-iii-tubulin-antibody-ep1569y-neuronal-marker-ab52623.html)
-Phospho-Ser133-CREB (Ser133) (Cell Signaling, 9198) previously validated in mouse lung section for IHC and in lysates from SK-N-MC cells for WB as stated by the manufacturers on their website (https://www.cellsignal.com/products/primary-antibodies/phospho-creb-ser133-87g3-rabbit-mab/9198)
-c-FOS (Abcam, ab190289) previously validated in HeLa cell lysate as stated by the manufacturers on their website (https://www.abcam.com/products/primary-antibodies/c-fos-antibody-bsa-free-ab190289.html)
-alpha-Tubulin-HRP (Abcam, ab40742) previously validated in HeLa cell Lysate, NIH/3T3 cell lysate and in brain (rat) tissue lysate as stated by the manufacturers on their website (https://www.abcam.com/products/primary-antibodies/hrp-alpha-tubulin-antibody-

dm1a-loading-control-ab40742.html)
-HES1 (Cell Signaling, 11988S) previously validated in mouse spinal cord section as stated by the manufacturers on their website (https://www.cellsignal.com/products/primary-antibodies/hes1-d6p2u-rabbit-mab/11988)
-MCT1 (Sigma, ab1286-I) previously validated by the manufacturers.
-GPX4 (Abcam, ab125066) previously validated in Human embryonic kidney epithelial cell, lung carcinoma epithelial cell whole cell lysate and mouse lysates by the manufacturers on their website (https://www.abcam.com/products/primary-antibodies/glutathione-peroxidase-4-antibody-epncir144-ab125066.html).
-4-hne (Abcam, ab46545) previously validated in frozen mouse cardiac tissue by the manufacturers on their website (https://www.abcam.com/products/primary-antibodies/4-hydroxynonenal-antibody-ab46545.html).
-LC3B (Cell Signaling, 43566) previously validated in HCT 116 and HCT 116 LC3B knockout cells by the manufacturers on their website (https://www.cellsignal.com/products/primary-antibodies/lc3b-e7x4s-xp-rabbit-mab/43566).
-Hsp90 (BD, 610418) previously validated by the manufacturers.
-FLAG (Cell Signaling, 2368) previously validated in COS or 293T cells by the manufacturers on their website (https://www.cellsignal.com/products/primary-antibodies/dykddddk-tag-antibody-binds-to-same-epitope-as-sigma-s-anti-flag-m2-antibody/2368)
-Ki67 (Abcam, ab15580) previously validated in mouse spleen formalin fixed paraffin embedded tissue section by the manufacturers on their website (https://www.abcam.com/products/primary-antibodies/ki67-antibody-ab15580.html).
-Cleaved Caspase 3 (Cell Signaling, 9579) previously validated in paraffin-embedded Jurkat cell pellets by the manufacturers on their website (https://www.cellsignal.com/products/primary-antibodies/cleaved-caspase-3-asp175-d3e9-rabbit-mab/9579).

Eukaryotic cell lines

Policy information about cell lines and Sex and Gender in Research

Cell line source(s)	Mouse SCLC (AD984LN_fl/adh; AF1165; AF3062C; AF1281m1; TP2031T2; AF3291LN_fl/adh; PRM2.1a), LUAD (KP1233, KP1234), PaNET (βTC-B6), and PDAC (MDM1402, MDM1403) cell lines were derived from GEMMs as previously described or as described in the method section. Human cell lines (HEK293T; NCI-H69_fl/adh; NCI-H889; NCI-H82; COR-L47; COR-L279) were either obtained from the Cell Services Core Facility depository or purchased from external sources including ATCC. PDX derived cell lines (MGH1505-1A) were obtained as described in the method section.
Authentication	Cell lines were authenticated by STR profiling.
Mycoplasma contamination	Cell lines were routinely tested for mycoplasma contamination.
Commonly misidentified lines (See ICLAC register)	No commonly misidentified cell lines were used in this study.

Animals and other research organisms

Policy information about studies involving animals; ARRIVE guidelines recommended for reporting animal research, and Sex and Gender in Research

Laboratory animals	Mouse. F1 mice (9-19weeks of age) were obtained by crossing 129S6/SvEv Tac males to C57Bl/6J females imported, respectively, from Taconic (Germantown, NY, USA) and Jackson Laboratories (Bar Harbor, ME, USA). NOD-SCID mice (Prkdcscid, JAX strain #001303, 7-12 weeks of age) were obtained from the Jackson laboratory. SCLC autochthonous mice (8-12 weeks old) harbor Trp53fl/fl, Rb1fl/fl, Rbl2fl/fl and Gt(ROSA)26Sortm14(CAG-tdTomato)Hze alleles (PRP130 model, described previously, see methods) or Trp53fl/fl, Rb1fl/fl, Rbl2fl/fl and Gt(ROSA)26Sortm1.1(CAG-tdTomato/GCaMP6f)Mdcah/J alleles (PRP130-Salsa6f model). PRP130 animals were on a mixed 129S5/C57Bl/6J background. PRM model (11 weeks old) was obtained from mice harbouring Trp53fl/fl, Rb1fl/fl, and Igs2tm1(CAG-Myc*T58A/luc)Wrey alleles (JAX strain #029971) on a C57Bl/6J background. 8–10-week-old Gt(ROSA)26Sortm14(CAG-tdTomato)Hze homozygous mice (Ai14 allele, JAX strain #007908) and NSG-GFP mice (JAX strain #021937) were also used.
Wild animals	No wild animals were used in this study
Reporting on sex	Age-, litter-, and sex-matched mice were randomly assigned to experimental groups.
Field-collected samples	No field-collected samples used in this study.
Ethics oversight	All procedures were conducted in accordance with the United Kingdom Animal (Scientific Procedures) Act 1986, approved by the Institutional Animal Welfare and Ethical Review Body (The Francis Crick Institute PPL Review Committee) and conducted under the authority of the UK Home Office approved Project License PP4103600, and approved by the Massachusetts Institute of Technology (MIT) Institutional Animal Care and Use Committee.

Note that full information on the approval of the study protocol must also be provided in the manuscript.

Plants

Seed stocks	NA
Novel plant genotypes	NA
Authentication	NA
